# Distinguishing homogeneous advanced oxidation processes in bulk water from heterogeneous surface reactions in organic oxidation

**DOI:** 10.1073/pnas.2302407120

**Published:** 2023-05-08

**Authors:** Ying-Jie Zhang, Jie-Jie Chen, Gui-Xiang Huang, Wen-Wei Li, Han-Qing Yu, Menachem Elimelech

**Affiliations:** ^a^Department of Environmental Science and Engineering, University of Science and Technology of China, Hefei 230026, China; ^b^Department of Chemical and Environmental Engineering, Yale University, New Haven, CT 06520

**Keywords:** Heterogeneous solid-water interface, homogeneous bulk water solution, reaction pathway, organic oxidation, Fenton-like catalytic

## Abstract

Clarifying the reaction pathways at the solid–water interface and in bulk water solution is of great significance for the design of heterogeneous catalysts for selective oxidation of organic pollutants. However, achieving this goal is daunting because of the intricate interfacial reactions at the catalyst surface. Herein, we unravel the origin of the organic oxidation reactions with metal oxide catalysts, revealing that the radical-based advanced oxidation processes (AOPs) prevail in bulk water but not on the solid catalyst surfaces. We show that such differing reaction pathways widely exist in various chemical oxidation (e.g., high-valent Mn^3+^ and MnO_X_) and Fenton and Fenton-like catalytic oxidation (e.g., Fe^2+^ and FeOCl catalyzing H_2_O_2_, Co^2+^ and Co_3_O_4_ catalyzing persulfate) systems. Compared with the radical-based degradation and polymerization pathways of one-electron indirect AOP in homogeneous reactions, the heterogeneous catalysts provide unique surface properties to trigger surface-dependent coupling and polymerization pathways of a two-electron direct oxidative transfer process. These findings provide a fundamental understanding of catalytic organic oxidation processes at the solid–water interface, which could guide the design of heterogeneous nanocatalysts.

## Significance

Heterogeneous catalytic oxidation technologies for water purification, such as Fenton and Fenton-like catalytic oxidation, involve intricate interfacial reactions at the solid catalyst surface and in bulk solution. To date, the difference in the reaction pathways on the catalyst surface and in bulk water solution has not been recognized. In this work, we reveal a widespread surface-dependent reaction pathway that is fundamentally different from the previously accepted pathway. We further elucidate the changes in reaction pathways as oxidizing species (e.g., Mn(III), •OH) detach from the catalyst surface to the aqueous solution. Our study provides new insights on heterogeneous chemical oxidation and catalytic oxidation reactions, potentially leading to the design of more efficient nanocatalysts.

Heterogeneous chemical oxidation reactions are ubiquitous in nature and play important roles in various chemical reaction systems associated with energy conversion, environmental remediation, and catalysis (1–4). Rapid advances in nanocatalysts have significantly improved the activity and stability of such reaction systems, opening the door for expanded applications. Nevertheless, one fundamental question remains: “How do the chemical reactions at the solid–water interface differ from those in bulk water?” (5).

Recent studies suggest that reactions occurring at the air–water interface and in bulk water solution differ drastically in reaction kinetics, with reaction rates based on the same reaction pathway varying greatly (5–7). We surmise that altered reaction kinetics compared to that in bulk water solution would also be expected at the solid–water interface of solid catalysts. However, since the nanocatalyst surface is greatly affected by factors such as morphology, atomic composition, atomic vacancies, crystal facets, and surface stress (8–10), the research blind spot for reaction kinetics is no longer the reaction rate, which is easy to measure, but rather the reaction pathway.

To date, the difference in reaction pathways between the solid–water interface and bulk water solution has not been fully revealed. For example, various Fenton and Fenton-like catalytic systems for organic pollutant oxidation have been reported—from the homogeneous Fe^2+^/hydrogen peroxide and Co^2+^/persulfate systems to the heterogeneous Fe- or Co-based solid catalyst/oxidant systems. In these reaction systems, the ions in solution were thought to play the same catalytic role as their counterparts on the heterogeneous catalyst surface and the removal of pollutants was considered to follow similar degradation/mineralization pathways (11–17). However, we recently revealed three unexpected functions, i.e., activation, stabilization, and accumulation, of catalyst surfaces for persulfate-triggered catalysis and organic pollutant oxidation (18), which inspire us to unravel the differences in reaction pathways between the solid–water interface and the bulk water solution. Clarifying the reaction pathways is of utmost importance for the design and optimization of heterogeneous catalytic oxidation processes and technologies.

In this work, we shed light on the different reaction pathways of organic oxidation at the solid catalyst surface and in aqueous solution. The reaction systems were constructed by using phenol (PhOH), a common compound in the chemical industry and a ubiquitous environmental pollutant (19–21), as a model organic compound, and a series of metal ions or metal oxide nanomaterials as the oxidants/catalysts. Manganese (Mn) is the second most abundant redox-active transition metal on earth with multiple valences [i.e., Mn(II), Mn(III), and Mn(IV)] in natural environments, such as the ocean floor, soils and sediments, and freshwater bodies (4, 22). Manganese oxides (MnO_X_) are one of the strongest naturally occurring oxidants with important roles in biogeochemical elemental cycles, soils, and water treatment (23–26). First, by using high-valent MnO_X_ solids (Mn_3_O_4_, Mn_2_O_3_, and MnO_2_) and high-valent Mn ions (Mn^3+^) as oxidants, the removal behavior, reaction pathways, and reaction sites of PhOH in heterogeneous and homogeneous oxidation systems were thoroughly investigated. We revealed an interface-dependent organic oxidation pathway, which has been considered an aqueous advanced oxidation process (AOP) in previous studies. Second, similar reaction pathway differences were extended to other heterogeneous catalytic oxidation systems (i.e., FeOCl catalyzing hydrogen peroxide and Co_3_O_4_ catalyzing persulfate) and the corresponding homogeneous ion catalytic oxidation (i.e., Fe^2+^ catalyzing hydrogen peroxide and Co^2+^ catalyzing persulfate) systems. Overall, our work clarified the reaction pathways in diverse homogeneous oxidation systems and their corresponding heterogeneous oxidation systems. These findings will inform and direct future studies on heterogeneous chemical (catalytic) oxidation processes, which are conventionally considered to proceed only by aqueous solution AOPs.

## Results

### PhOH Oxidation Pathway in MnO_X_ Heterogeneous Oxidation Systems.

**Fig. 1.**
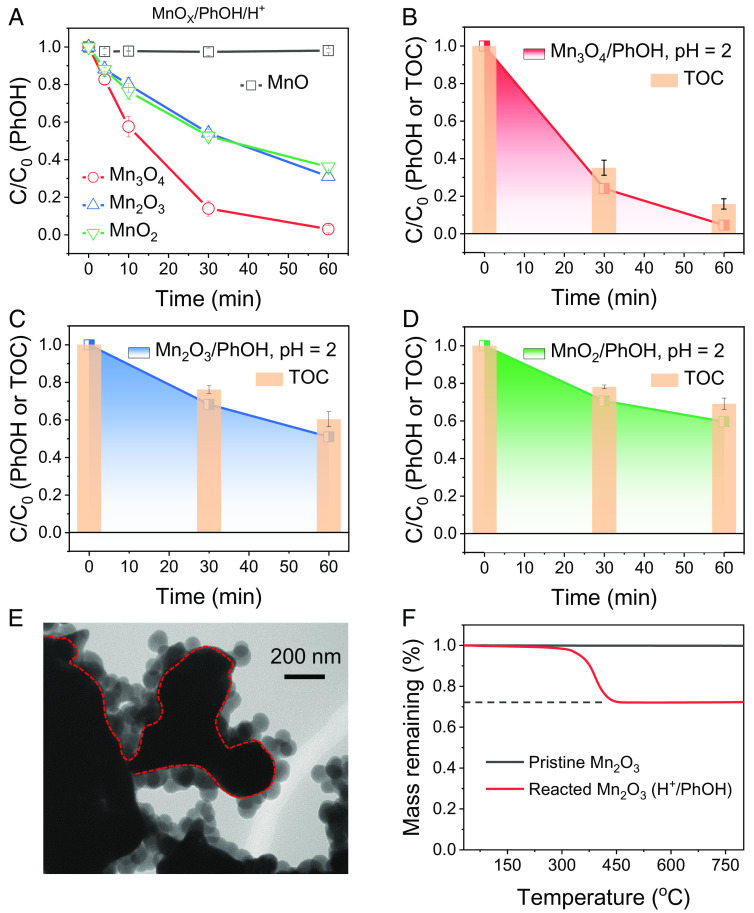
DOTP behavior of PhOH removal in the heterogeneous MnO_X_ oxidation systems. (*A*) Removal efficiency of PhOH in MnO, Mn_3_O_4_, Mn_2_O_3_, and MnO_2_ oxidation systems. (*B*–*D*) Residual PhOH concentration and the corresponding TOC removal in the (*B*) Mn_3_O_4_/PhOH, (*C*) Mn_2_O_3_/PhOH, and (*D*) MnO_2_/PhOH reaction processes. (*E*) TEM image of Mn_2_O_3_ after the reaction of Mn_2_O_3_/PhOH. (*F*) TGA curves of the pristine and reacted Mn_2_O_3_ in air (O_2_). Reaction conditions: [MnO_X_] = 0.6 g L^−1^, pH = 2, [PhOH] = 50 mg L^−1^ (for *A*) and [MnO_X_] = 0.2 g L^−1^, pH = 2, [PhOH] = 25 mg L^−1^ (for *B*–*D*).

We first investigated the chemistry of heterogeneous PhOH oxidation by various MnO_X_, including MnO, Mn_3_O_4_, Mn_2_O_3_, and MnO_2_ (*SI Appendix*, Figs. S1–S6). All the MnO_X_, except for MnO (which lacks high-valent Mn species), exhibited considerable activity toward PhOH oxidation (Fig. 1*A*), especially under acidic pH (*SI Appendix*, Figs. S2–S4), suggesting a possible role of high-valent Mn in such reactions. The oxidation of PhOH by high-valent Mn in MnO_X_ was supported by the pH changes of the reaction solution and Mn^2+^ leaching during the reaction. The increased solution pH values after the reaction (*SI Appendix*, Figs. S3*B*, S5 *D*–*F*, and S6 *D*–*F*) corresponded to Mn ion dissolution (Table 1), in which oxygen atoms are released into the aqueous solution with H^+^ consumption.

**Table 1.** Electron transfer numbers in the MnO_X_/PhOH-DOTP systems determined by monitoring the oxidative removal of PhOH and the reductive leaching of Mn^2+^ (pH = 2.0)

	Mn_3_O_4_/PhOH	Mn_2_O_3_/PhOH	MnO_2_/PhOH
DOTP ratios (%)	91.45	87.37	84.54
Removal amount of PhOH (mmol L^−1^)^^*^^	0.27	0.21	0.14
Leaching amount of Mn^2+^ (mg L^−1^)^†^	59.56	26.07	9.84
Obtained number of electrons (MnO_X_ → Mn^2+^)^‡^	0.67 (8/3→2)	1 (3→2)	2 (4→2)
Obtained concentration of electrons (mmol L^−1^)^§^	0.72	0.47	0.36
Electron transfer numbers in MnO_X_/PhOH^¶^	2.67	2.24	2.57

^*^The data were obtained by UHPLC in the MnO_X_/PhOH reaction systems.

^†^The data were obtained by ICP-MS in the MnO_X_/PhOH reaction systems.

^‡^The data were calculated according to the valence state changes of Mn. Taking Mn_3_O_4_ → Mn^2+^ as an example, the average valence states of Mn in Mn_3_O_4_ and Mn^2+^ are 8/3 and 2, respectively. Leaching 1 mol Mn^2+^ will obtain 0.67 (i.e., 8/3-2) mol of electrons.

^§^The data were obtained according to the formula: d = b/55*c.

^¶^The data were obtained according to the formula: e = d/a.

To analyze the oxidation pathways of PhOH in the Mn_3_O_4_/PhOH, Mn_2_O_3_/PhOH, and MnO_2_/PhOH systems, the removal efficiencies of total organic carbon (TOC) in these reaction processes were evaluated. The aqueous TOC was synchronously removed with PhOH at high efficiency in all the test groups (Fig. 1 *B*–*D*), which differs from the PhOH degradation behavior in AOPs (20). To clarify such an unusual phenomenon of aqueous TOC removal, we examined the surface characteristics of MnOx before and after the reaction. The transmission electron microscopy (TEM) images showed lighter contrast layers on the MnO_X_ nanosurface (Fig. 1*E* and *SI Appendix*, Figs. S7 and S8). In addition, the measurement of the mass loss of organics by thermal gravimetric analysis (TGA) also suggests that the majority of PhOH in the aqueous solution was transferred and accumulated onto the MnO_X_ nanosurface in the oxidation processes (Fig. 1*F* and *SI Appendix*, Fig. S9), with transfer efficiencies of 91.45% (Mn_3_O_4_), 87.37% (Mn_2_O_3_), and 84.54% (MnO_2_) (Table 1). These results suggest that a direct oxidative transfer process (DOTP), rather than AOP, may predominate in the high-valent MnO_X_/PhOH oxidation systems, during which organics are not decomposed, but oxidized and accumulate on catalyst surfaces.

To further decipher the pathways of PhOH oxidation at the MnO_X_ surface, we identified the molecular structures of the surface-accumulated products. Taking the Mn_2_O_3_/PhOH system as an example, we tried to elute the reacted Mn_2_O_3_ with ethanol and toluene to dissolve the surface-accumulated product but failed to do so (*SI Appendix*, Fig. S10), implying that the product might be in a cross-linked state. Having three active sites in its molecular structure (i.e., the ortho- and para-positions of the hydroxyl group), PhOH is readily oxidized and forms a network-like cross-linked polymer that is insoluble in organic solvents (18). If this is the case, the formation of the cross-linked product would be prevented when two of the three active sites in the reactant are sheltered. For validation, we conducted the hypothetico-deductive experiment using 2,6-dimethyl-phenol (2,6-M-PhOH) as the reactant, which has only one active hydrogen site preserved in its molecular structure.

#### Hypothesis.

The 2,6-M-PhOH oxidation on the Mn2O3 surface would proceed by the same pathway as that of PhOH but form a noncross-linked product that can be readily dissolved in organic solvents.

#### Deduction.

The Mn_2_O_3_ reacted with 2,6-M-PhOH was eluted with ethanol and toluene step by step following the same procedures used in the Mn_2_O_3_/PhOH system.

#### Deduction results.

The aqueous 2,6-M-PhOH and TOC were transferred and accumulated onto the Mn_2_O_3_ surface after the reaction, and the surface-accumulated products could be thoroughly washed off by ethanol and toluene (*SI Appendix*, Fig. S11). These results further support the predominance of the DOTP pathway in both the Mn_2_O_3_/PhOH and Mn_2_O_3_/2,6-M-PhOH systems.

**Fig. 2.**
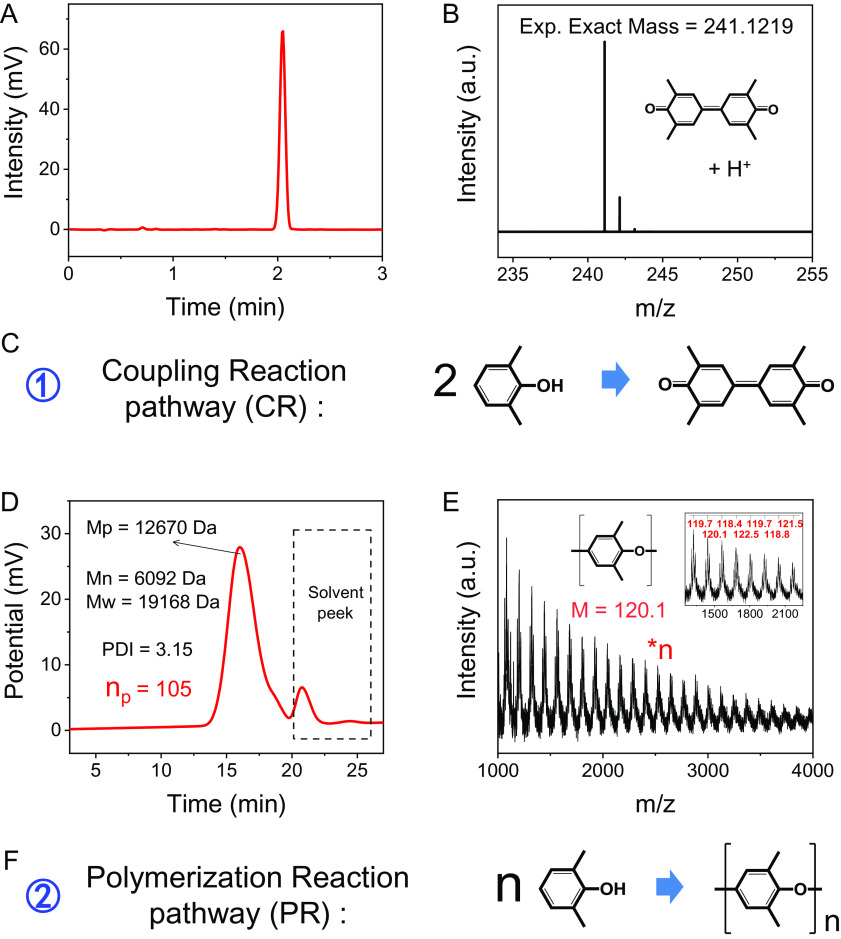
Oxidation pathway analyses of the phenol (2,6-MPhOH) removal process in the Mn_2_O_3_/2,6-MPhOH-DOTP system. (*A* and *B*) High-performance liquid chromatogram (*A*) and high-resolution mass spectrum (*B*) of the products on the reacted Mn_2_O_3_ washed off by ethanol. The theoretical exact mass of 3,3,5,5-tetramethyl-4,4′-biphenyl-quinone (positive ion acquisition mode, +H) is 241.1228, consistent with the experimental value in *B* (i.e., 241.1219, error <2 ppm). (*C*) Schematic of the surface coupling reaction pathway. (*D* and *E*) Gel permeation chromatogram (*D*) and MALDI-TOF-MS spectrum (*E*) of the products on the reacted Mn_2_O_3_ washed off by toluene. The *Inset* in *E* is a partially enlarged view of the MALDI-TOF-MS spectrum, in which the mean mass interval of 120.1 matches the polymeric unit of poly(2,6-dimethyl-1,4-phenylene oxide) (PPO). (*F*) Schematic of the surface polymerization reaction pathway. Exp., experimental; M_p_, peak molecular weight; M_n_, number average molecular weight; M_w_, weight average molecular weight; N, degree of polymerization; N_p_, degree of polymerization at the peak molecular weight.

To provide direct evidence of the DOTP pathway, we identified the dissolved 2,6-M-PhOH oxidation products by liquid chromatography mass spectrometry (LCMS), gel permeation chromatography (GPC), and matrix-assisted laser desorption/ionization time-of-flight mass spectrometry (MALDI-TOF-MS). The results indicate that the substances dissolved in ethanol were biphenyl quinone compounds (i.e., 3,3′,5,5′-tetramethyl-diphenoquinone) (Fig. 2 *A* and *B*), while those dissolved in toluene were identified as chain-like polyphenyl ethers with a peak molecular weight of ~4,635 Da (Fig. 2 *D* and *E*). The former could be generated by the C–C coupling of 2,6-M-PhOH (i.e., Fig. 2*C*) (27, 28), and the latter may be derived from the C–O polymerization of 2,6-M-PhOH (Fig. 2*F*) (29, 30). Therefore, the PhOH oxidation in the high-valent MnO_X_ systems proceeds by both the coupling reaction (CR) and polymerization reaction (PR) pathways, two types of DOTP pathways.

### Dependence of the DOTP Pathway on the Solid–Water Interface.

**Fig. 3.**
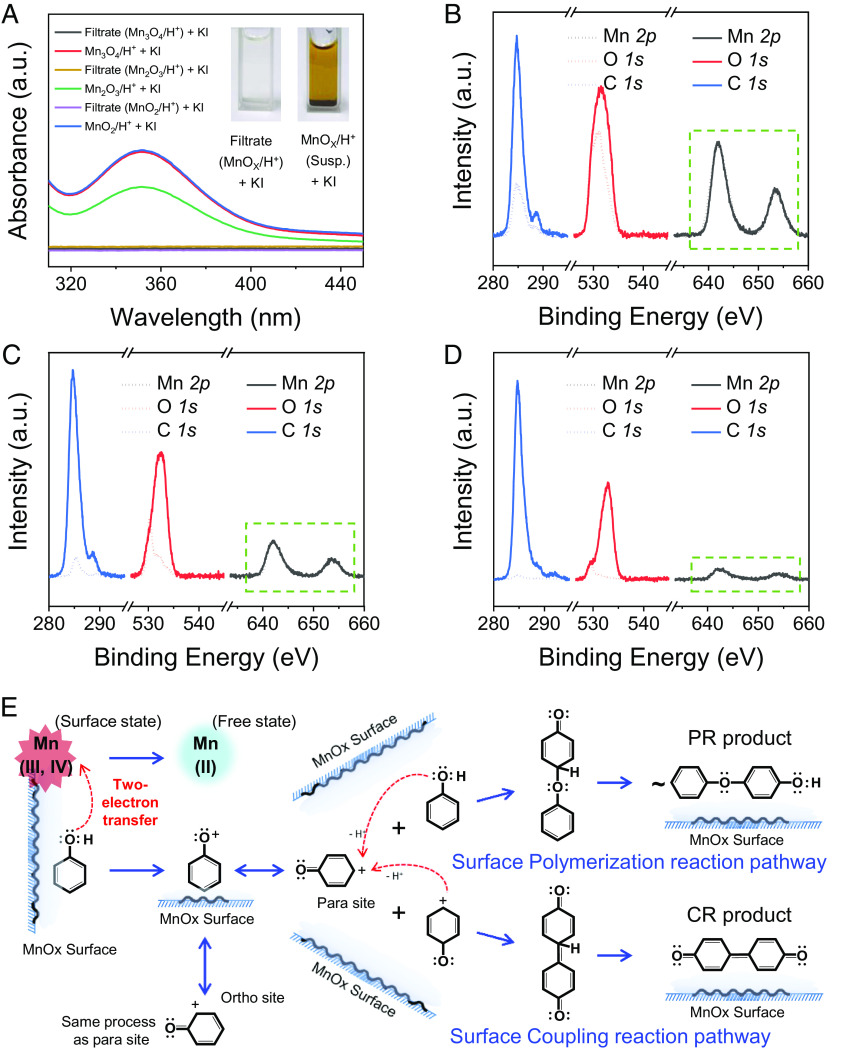
Surface-dependent elementary reaction pathways of DOTP in the MnO_X_ solid–aqueous interface oxidation systems. (*A*) Ultraviolet visible absorption spectra of the KI oxidation reaction in the filtrate and suspension of MnO_X_/H^+^. The inset in A shows the optical photographs of KI + filtrate and KI + suspension. (*B*–*D*) XPS spectra of the pristine and reacted (*B*) Mn_3_O_4_, (*C*) Mn_2_O_3_, and (*D*) MnO_2_. The dashed lines represent the pristine MnO_X_, while the solid lines represent the reacted MnO_X_. The signal intensities in the XPS spectra of the pristine and reacted MnO_X_ were normalized by that of Mn *2p*. The lack of change in the Mn *2p* peak shape and peak position indicates that the surface valence states of Mn after the reaction were the same as those of pristine MnO_X_. (*E*) Proposed elementary reaction pathways of PhOH (from reactants to the surface coupling and polymerization reaction products) in the MnO_X_ solid–aqueous interface oxidation systems. Susp., suspension (i.e., solution containing MnO_X_ particles); PR, polymerization reaction; CR, coupling reaction.

Next, we performed potassium iodide (KI) oxidation experiments to further identify the PhOH reaction sites in the MnO_X_ oxidation system (Mn_3_O_4_, Mn_2_O_3_, and MnO_2_). We surmised that dissolved high-valent Mn ions from MnO_X_ under acidic conditions, if present, would efficiently oxidize KI, and this chromogenic reaction can be easily detected by Ultraviolet visible (UV–Vis) absorption spectroscopy. However, no optical or spectral changes in the supernatant solution (i.e., the filtrate of the MnO_X_/H^+^ suspension) were observed after mixing with KI (Fig. 3*A*), suggesting that no dissolution of free high-valent Mn ions occurred in the acidic Mn_3_O_4_, Mn_2_O_3_, and MnO_2_ nanoparticle suspensions. Interestingly, when KI was directly added to these acidic MnO_X_ suspensions, the chromogenic reaction occurred immediately (Fig. 3*A*). Apparently, PhOH oxidation occurred predominantly on the nanosurface of high-valent MnO_X_ (i.e., at the solid–water interface) instead of in the bulk water solution.

Since Mn_3_O_4_, Mn_2_O_3_, and MnO_2_ do not dissolve in an acidic environment without redox reactions (*SI Appendix*, Tables S1 and S2), the released Mn ions in the high-valent MnO_X_/PhOH reaction systems should result from MnO_X_ reduction. The X-ray photoelectron spectra (XPS) of Mn_3_O_4_, Mn_2_O_3_, and MnO_2_ before and after the reaction show that the valence states (reflected by the peak shapes and positions) of surface Mn did not change during the DOTP reaction (Fig. 3 *B*–*D* and *SI Appendix*, Fig. S12), suggesting that the formed reduced-state Mn (i.e., Mn^2+^) during the DOTP reaction was completely released into the bulk solution. This result is consistent with the high solubility of MnO in acidic environments (*SI Appendix*, Table S1). Therefore, the leached concentration of Mn^2+^, together with the removal amount of PhOH in the solution, could be used to estimate the electron transfer number of the DOTP reaction in the high-valent MnO_X_/PhOH systems. Our results show that the electron transfer numbers in all these reaction systems were close to two (Table 1), indicating that the DOTP reactions of PhOH proceed by two-electron direct reaction pathways at the high-valent MnO_X_ surface. The detailed elementary reaction pathways are depicted in Fig. 3*E*.

### PhOH Oxidation Pathway in Mn^3+^ Homogeneous Oxidation System.

**Fig. 4.**
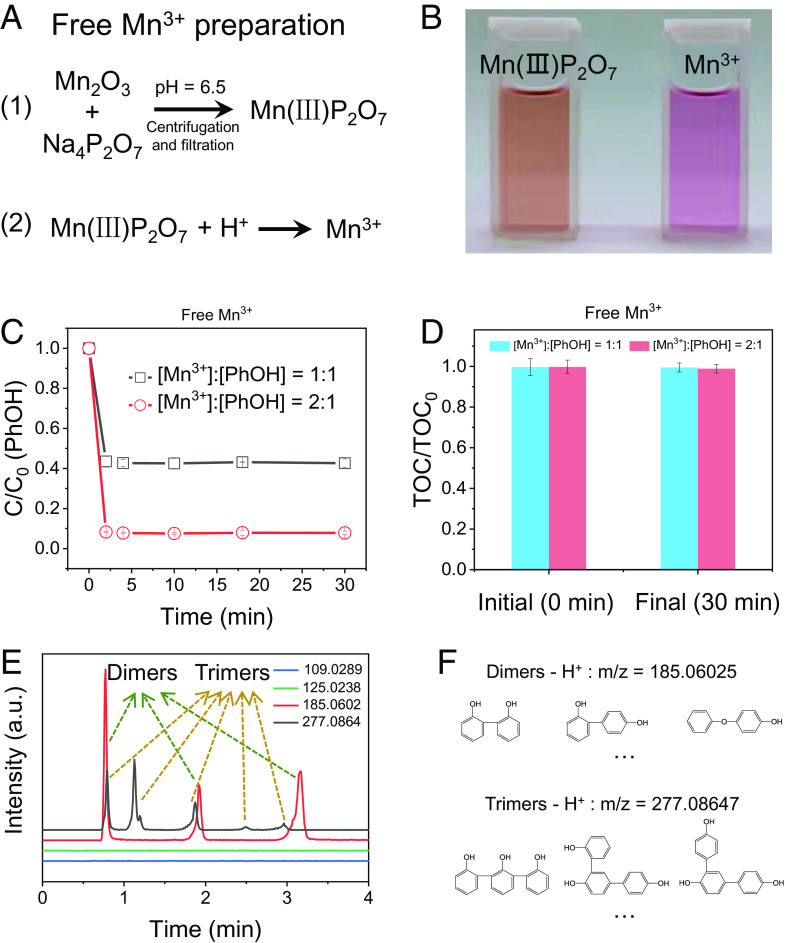
Preparation of free Mn^3+^ and the AOP behaviors of Mn^3+^-dominated PhOH removal in bulk solution. (*A*) Preparation processes of free Mn^3+^, including coordination dissolution (Step 1) and acidification dissociation (Step 2). (*B*) Optical photographs of the Mn(III)P_2_O_7_ solution and the free Mn^3+^ solution. (*C*) PhOH removal efficiency under different dosages of free Mn^3+^. (*D*) TOC changes in *C*. (*E*) Product separation and identification in the Mn^3+^-dominated PhOH oxidative removal process by LC-MS. (*F*) Structures and m/z (negative ion acquisition mode, −H) of the products in *E*.

To further confirm that the DOTP reaction of PhOH and solid Mn(III, IV) (the reactive species in high-valent MnO_X_) is indeed a surface-dependent pathway and to elucidate the underlying mechanisms, we constructed a homogeneous PhOH oxidation system with dissolved-state Mn(III) (i.e., free Mn^3+^ in water) for comparison. Here, free Mn^3+^ was obtained by two consecutive steps: coordination dissolution and acidification dissociation (Fig. 4*A*). First, Mn_2_O_3_ was dissolved into bulk water with the help of pyrophosphate (PP), a strong but nonredox-active ligand (11, 31), to form a homogeneous Mn(III)–PP bulk solution (Fig. 4*B* and *SI Appendix*, Fig. S13*A*). The oxidizing ability of Mn(III) in this Mn(III)–PP complex is restricted by the ligands, as evidenced by its inability to oxidize KI (*SI Appendix*, Fig. S13*B*). Then, Mn(III)–PP was acidified with H_2_SO_4_, by which process Mn(III) was freed to bulk water via proton (H^+^) substitution (Fig. 4*B* and *SI Appendix*, Fig. S13*B*). The amount of obtained free Mn^3+^ was quantified by KI oxidation chromogenic reaction (a typical one-electron transfer reaction) (*SI Appendix*, Fig. S14*A*) following an analogous procedure used for persulfate [including peroxymonosulfate (PMS) and peroxydisulfate (PDS)] concentration determination (24, 32).

The PhOH removal behavior in the Mn^3+^ homogeneous oxidation system was explored qualitatively and quantitatively. At low Mn^3+^ dosages (i.e., no more than twice the molar amount of PhOH), the TOC in bulk water remained almost unchanged, although PhOH was rapidly and largely removed (Fig. 4 *C* and *D* and *SI Appendix*, Fig. S14*B*). This result indicates that the oxidation products remained in the bulk water, which is consistent with the PhOH removal behavior in AOPs (20). Subsequently, we separated the Mn^3+^/PhOH reaction product from the solution by ultrahigh-performance liquid chromatography (UHPLC). High-resolution mass spectral analysis of the separated and recovered product showed the formation of both dimers and trimers of PhOH (Fig. 4 *E* and *F* and *SI Appendix*, Fig. S15), which should result from the oligomerization of phenoxy radicals (23, 33). Therefore, the PhOH reaction pathways in the Mn^3+^ homogeneous oxidation system (dominated by radical polymerization reaction, a typical AOP) differed fundamentally from those in the DOTP-based heterogeneous MnO_X_ reaction system. These findings clarify the long-standing misunderstandings about AOPs in MnO_X_ oxidation systems (17, 25, 34, 35) and identify the change of reaction pathways as oxidizing species [i.e., Mn(III, IV)] detach from the heterogeneous surface to aqueous solution.

### Universality of the Pathways in Fenton and Fenton-Like Catalytic Oxidation Systems.

**Fig. 5.**
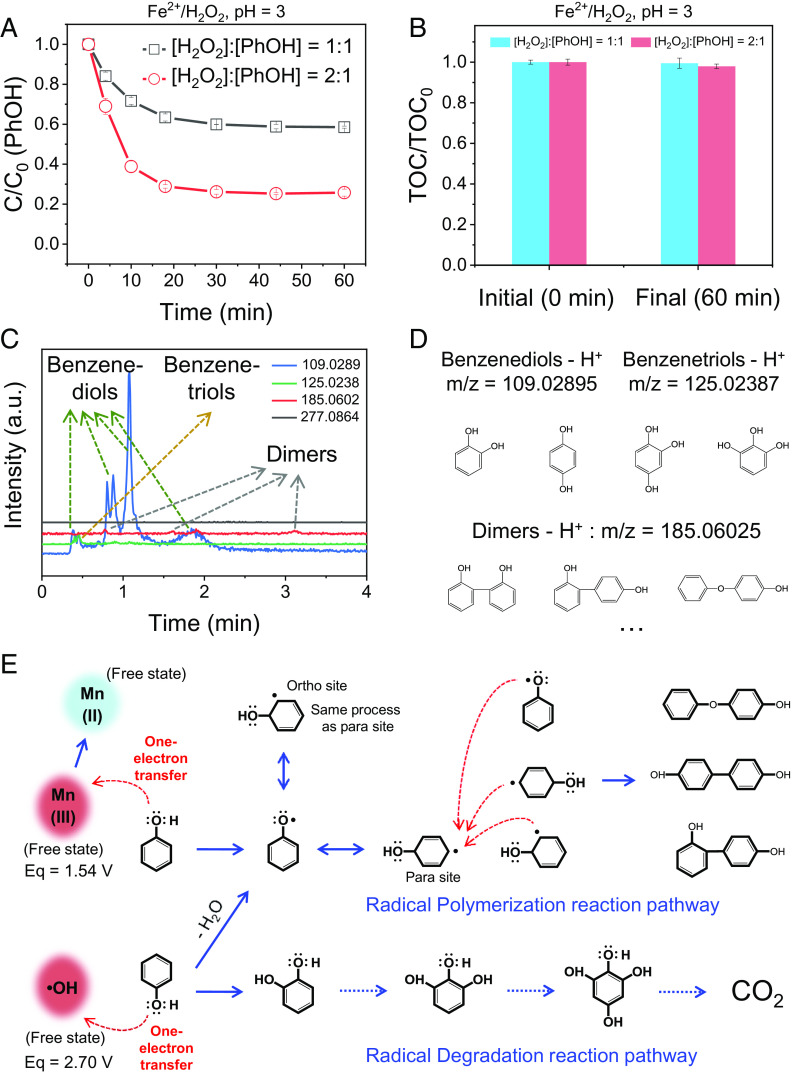
Free •OH-dominated PhOH oxidative removal process and elementary reaction pathways of AOP in bulk solution. (*A*) PhOH removal efficiency in the Fenton system (Fe^2+^/H_2_O_2_, the typical •OH-dominated AOP system). (*B*) TOC changes in *A*. (*C*) Product separation and identification in the •OH-dominated PhOH oxidative removal process by LC-MS. (*D*) Structures and m/z (negative ion acquisition mode, −H) of the products in *C*. (*E*) Proposed elementary reaction pathways of PhOH (from reactants to the radical degradation and polymerization products) in the homogeneous oxidation systems of Mn^3+^ and •OH in bulk solution.

Similar to the homogeneous Mn^3+^ oxidation system, the classical Fe^2+^/H_2_O_2_ Fenton catalytic oxidation system is dominated by a radical pathway in organic pollutant degradation/mineralization (7, 36, 37). As expected, the removal behavior of aqueous PhOH and TOC in the homogeneous Fe^2+^/H_2_O_2_ reaction system was analogous to that in the Mn^3+^ oxidation system: Rapid PhOH removal but no obvious TOC removal from the bulk water was observed at low H_2_O_2_ dosage (Fig. 5 *A* and *B*). The reaction products in the Fe^2+^/H_2_O_2_ reaction system were mainly benzenediols and benzenetriols [the free-radical adducts of PhOH (16, 20)] and dimers of PhOH (oligomerization of phenoxy radicals), which resulted from the radical degradation and polymerization of PhOH, respectively (23, 33) (both belong to AOPs) (Fig. 5 *C* and *D* and *SI Appendix*, Fig. S16). Compared with the Mn^3+^ oxidation system, the occurrence of the PhOH radical degradation pathway in the Fe^2+^/H_2_O_2_ catalytic oxidation system might be attributed to the higher oxidizing ability of hydroxyl radicals (standard oxidation potential 2.7 V) than that of Mn^3+^ (oxidation potential 1.54 V) (38–40).

The results from the homogeneous reaction systems of Mn^3+^/PhOH and hydroxyl radical/PhOH in bulk water indicate that the oxidation pathway of PhOH was an indirect oxidation process. In these systems, the oxidizing species of Mn^3+^ and hydroxyl radicals were first generated from high-valent MnO_X_ and H_2_O_2_, which involved a one-electron-transfer intermediate step. Then, the generated oxidizing species underwent radical degradation and radical polymerization reactions, depending on their oxidation potential. The elementary reaction pathways of PhOH oxidized by Mn^3+^ and hydroxyl radicals are detailed in Fig. 5*E*. Such AOP reaction pathways differ fundamentally from the abovementioned DOTP in heterogeneous oxidation systems.

**Fig. 6.**
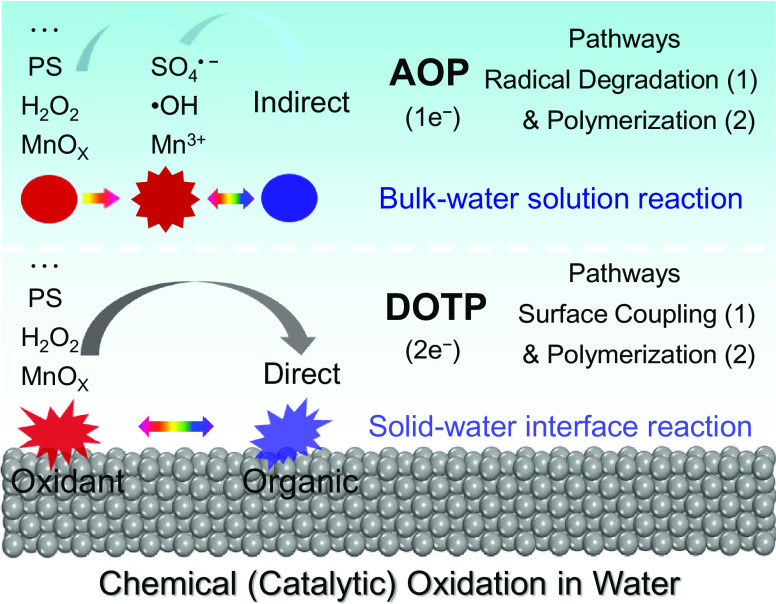
Schematic illustration of the features of organic oxidation pathways in bulk water and at solid–water interface. At oxidative solid–water interfaces, such as in the high-valent MnO_X_, solid–catalyst/H_2_O_2_, and solid–catalyst/persulfate systems, the 2-electron direct oxidation and surface-dependent coupling and polymerization pathways occur. While in bulk water, the corresponding oxidative species of Mn^3+^, •OH, and SO_4_^•−^ should be produced first from the high-valent MnO_X_, H_2_O_2_, and persulfate, and then the 1-electron indirect oxidation and radical-mediated degradation and polymerization pathways occur. H_2_O_2_, hydrogen peroxide; PS, persulfate.

Corresponding to the homogeneous Fenton system in bulk water, the oxidation pathways of PhOH in the heterogeneous Fenton system were also explored by using FeOCl [the most efficient Fe-based nanocatalyst (14, 41)] as a representative heterogeneous catalytic material. The results show that FeOCl could effectively catalyze H_2_O_2_ to oxidize PhOH (*SI Appendix*, Figs. S17 and S18 *A* and *B*). Even at a low dosage ratio of H_2_O_2_ to PhOH (i.e., 2:1), the TOC and chemical oxygen demand (COD) removal efficiencies were still at high levels similar to those in the MnO_X_ oxidation system (*SI Appendix*, Fig. S18 *C* and *D*), suggesting that the DOTP pathway might also dominate the PhOH oxidation in the FeOCl/H_2_O_2_ heterogeneous Fenton system. This was verified by product analysis, which showed the accumulation of PhOH oxidizing on the reacted FeOCl surface (formed from surface-dependent coupling and polymerization pathways) (*SI Appendix*, Figs. S19 and S20). Similar results were found for the PhOH oxidation pathways in the Fenton-like process of Co^2+^/PMS (homogeneous catalytic system) and Co_3_O_4_/PMS (heterogeneous catalytic system) (*SI Appendix*, Figs. S21 *A* and *B*, S22, and S23). Together, these results indicate that the difference in the organic oxidation pathways between the solid–water interface and bulk water solution exists universally in diverse chemical (catalytic) oxidation systems (Fig. 6).

## Discussion

Our systematic analysis of PhOH oxidation in homogeneous catalytic oxidation systems and their counterpart heterogeneous catalytic oxidation systems clarified the changes in the reaction pathways as oxidizing species (i.e., high-valent Mn, hydroxyl radical, and sulfate radical) detach from the heterogeneous surface to aqueous solution. In the homogeneous Mn^3+^ oxidation system as well as Fe^2+^/H_2_O_2_ (Fenton) and Co^2+^/PMS (Fenton-like) catalytic oxidation systems, organics are mainly removed by AOPs via radical degradation and radical polymerization pathways (one-electron indirect oxidation). In these reaction systems, the oxidants must first be activated to produce highly reactive intermediates (such as ROS) via one-electron pathway, with the latter primarily responsible for the pollutant degradation or polymerization. Such radical-mediated reactions are inevitably energy and chemical intensive. These reactions also suffer from incomplete pollutant removal from water and generation of hazardous by-products, which limit their application in water purification.

In contrast, the heterogeneous reaction systems were dominated by surface-dependent coupling and polymerization reactions via two-electron direct oxidation pathway (i.e., DOTP reaction), which allows nondegradative yet highly efficient, complete removal of organic pollutants from water. Specifically, the oxidants on nanocatalyst surfaces directly oxidize the organic pollutants via a two-electron reaction pathway without an ROS intermediate step. In such processes, the oxidized pollutant intermediates are stabilized by the catalyst surface and spontaneously undergo surface coupling and polymerization reactions with other intermediates or pollutant molecules. Their products accumulate in situ on the catalyst surface and the aqueous pollutants are completely removed. The entire process requires much less oxidants and leaves almost no residual by-products in water. Such surface-dependent DOTP reactions were universally found in the high-valent MnO_X_ (i.e., Mn_3_O_4_, Mn_2_O_3_, and MnO_2_) oxidation systems as well as solid FeOCl/H_2_O_2_ (heterogeneous Fenton) and Co_3_O_4_/persulfate (heterogeneous Fenton-like) catalytic oxidation systems, suggesting a great potential for water purification applications.

Distinguishing the difference between homogeneous and heterogeneous oxidation (i.e., the vast difference in pollutant removal effect and kinetics caused by different reaction pathways) would allow us to develop more efficient, economically affordable water treatment nanotechnologies. In the long term, environmentally benign, simple, efficient, and low-cost heterogeneous chemical oxidation (catalytic oxidation) technologies will be highly desired for the purification of organic-polluted wastewaters. Therefore, our findings shed light on a long-lasting misunderstanding of chemical oxidation/catalytic oxidation processes at solid–water interfaces, which may guide the development of more efficient and sustainable water purification systems.

## Materials and Methods

### Materials.

Nano MnO (99.5%), Mn_3_O_4_ (97%), Mn_2_O_3_ (99%), MnO_2_ (99%), nano cobalt(II, III) oxide (Co_3_O_4_, 99.5%), iron(III) chloride hexahydrate (FeCl_3_·6H_2_O, 99%), PhOH (99.5%), 2,6-dimethylphenol (2,6-M-PhOH, 99.5%), potassium iodide (KI, 99.5%), and potassium dichromate (K_2_Cr_2_O_7_, 99.9%) were purchased from Aladdin Co., China. Tetrasodium PP decahydrate (Na_4_P_2_O_7_·10H_2_O, 99%), concentrated H_2_SO_4_ (GR), hydrogen peroxide (H_2_O_2_, 30%), iron sulfate heptahydrate (FeSO_4_·7H_2_O, 99%), potassium persulfate (PDS, 99.5%), ascorbic acid (99.5%), ethanol, toluene, tetrahydrofuran, and acetone were purchased from Shanghai Chemical Reagent Co., China. Silver sulfate (Ag_2_SO_4_) and mercuric sulfate (HgSO_4_) were purchased from Shanghai Macklin Biochemical Co., China. PMS (2KHSO_5_·KHSO_4_·K_2_SO_4_, 4.5% active oxygen) was purchased from Beijing J&K Co., China. Mn(III)-PP stock solutions were prepared according to a previously reported protocol (11, 42). Specifically, 2 g Mn_2_O_3_ was first added to 100 mL 40 mM Na_4_P_2_O_7_·10H_2_O (PP) solution. Then, the pH of the suspension was adjusted to 6.5 with H_2_SO_4_. After continuous stirring at ambient temperature for 5 d, the suspension was centrifuged and filtered to obtain the Mn(III)–PP stock solution. Nano FeOCl was also synthesized according to a reported procedure (14, 43). Briefly, 2 g FeCl_3_·6H_2_O powder was first heated and annealed at 220 °C for 2 h. Then, the obtained FeOCl powder was washed with acetone and water. The pH adjustment and acidification of the reaction solution were all performed by using H_2_SO_4_ (nonredox activity and no coordination ability). Unless otherwise specified, all chemicals were used as received without further purification.

### Batch Experiments.

For the batch experiments of the PhOH reaction in MnO_X_ (MnO, Mn_3_O_4_, Mn_2_O_3_, and MnO_2_) oxidation systems, a dose of MnO_X_ powder was first added to 40 mL PhOH solution at varying concentrations. After 5-min ultrasonic dispersion, the suspension was stirred for 15 min to establish adsorption–desorption equilibrium (the initial pH ~ 6). Then, an aliquot of H_2_SO_4_ was added to the suspension to adjust the pH and initiate the reaction. For the batch experiments of the PhOH reaction in Mn^3+^ oxidation systems, Mn(III)–PP stock solution was first added to 40 mL PhOH solution at predetermined concentrations. Then, an aliquot of H_2_SO_4_ was added to the mixture to free Mn^3+^ and initiate the reaction.

For the batch experiments of the PhOH reaction in the Fe^2+^/H_2_O_2_, FeOCl/H_2_O_2_, Co^2+^/PMS, and Co_3_O_4_/PMS catalytic oxidation systems, the catalysts of Fe^2+^, FeOCl, Co^2+^, and Co_3_O_4_ were first added to the PhOH solution at preset concentrations. Then, the reactions were initiated by adding the oxidants (a certain amount of H_2_O_2_ or PMS).

In the above reaction systems, the concentrations of PhOH, TOC, and COD over time were measured to monitor the reaction progress. For the reaction systems requiring TEM and TGA characterizations, the concentration of reactants was usually increased 10 ~ 20 times. All experiments were conducted in triplicate, and error bars are expressed as the arithmetic mean ± SD.

### Validation of Surface-Dependent Effect.

The surface-dependent effect of the DOTP pathway was validated by KI oxidation and chromogenic reactions in high-valent MnOX/PhOH reaction systems. Specifically, a certain amount of Mn_3_O_4_, Mn_2_O_3_, and MnO_2_ powders was first added to the dilute sulfuric acid solution (pH = 2.0) and stirred with ultrasonication for 60 min. Then, the suspension was divided into two portions. One portion of the suspension was centrifuged and filtrated to obtain the bulk solution, which was subsequently mixed with KI solution and detected with UV–Vis absorption spectroscopy (2450, SHIMADZU Co., Japan). Another portion was directly mixed with KI solution, and the supernatant was detected with the same UV–Vis absorption spectroscopy.

### Measurement of Mn^3+^ Concentration.

The Mn^3+^ concentration in the Mn^3+^ stock solution was quantified by KI oxidation and chromogenic reaction. Specifically, 1 mL Mn(III)–PP stock solution (diluted if necessary) was first mixed with 1 mL KI solution (100 g L^−1^, containing 5 g L^−1^ NaHCO_3_). Then, a certain amount of H_2_SO_4_ was added to the mixture to free Mn^3+^ and initiate KI oxidation and chromogenic reactions. After 30 min of reaction, the mixture was analyzed by a UV–Vis absorption spectrometer (2450, SHIMADZU Co., Japan) at 396 nm. For absolute quantification of Mn^3+^ (the one-electron transfer oxidant), the standard substance PDS (the two-electron transfer oxidant) was used to calibrate the standard curve of oxidant concentration versus absorbance intensity. The procedure used in the PDS/KI oxidation and chromogenic reaction was the same as that used for Mn^3+^/KI.

### Analytical Methods.

The qualitative and quantitative analyses, including PhOH, TOC, and COD concentrations, hypothetico-deductive experiments, and characterization techniques (X-ray powder diffraction (XRD), TEM, TGA, XPS, inductively coupled plasma-mass spectrometer (ICP-MS), LC–MS, GPC, MALDI-TOF-MS), are available in *SI Appendix*.

## Supplementary Material

Appendix 01 (PDF)

## Data Availability

All study data are included in the article and/or *SI Appendix*.
