# Supplementary material for: Distinguishing homogeneous advanced oxidation processes in bulk water from heterogeneous surface reactions in organic oxidation
Source: Proc Natl Acad Sci U S A. 2023 May 8;120(20):e2302407120. doi: 10.1073/pnas.2302407120 (PMC10193935; doi:10.1073/pnas.2302407120)
Supplement: Supplementary file 1 — Appendix 01 (PDF) [file pnas.2302407120.sapp.pdf]

Supplementary Information

Distinguishing homogeneous advanced oxidation processes in bulk water from heterogeneous surface reactions in organic oxidation

Ying-Jie Zhang[1], Jie-Jie Chen[1], Gui-Xiang Huang[1], Wen-Wei Li[1]*, Han-Qing Yu[1]*, Menachem Elimelech[2]*

[1]Department of Applied Chemistry, University of Science and Technology of China, Hefei, 230026, China

[2]Department of Chemical and Environmental Engineering, Yale University, New Haven, CT 06520, USA

***Corresponding authors:**

Prof. Wen-Wei Li, E-mail: wwli@ustc.edu.cn

Prof. Han-Qing Yu, E-mail: hqyu@ustc.edu.cn

Prof. Menachem Elimelech, E-mail: menachem.elimelech@yale.edu

This PDF file includes:

Supplementary Methods

Supplementary Figures 1 to 23

Supplementary Table 1 to 2

Supplementary Methods

Quantification of phenols

In the batch experiments, one milliliter of the suspension/solution was withdrawn and quenched with ascorbic acid solution at given time intervals once the reaction was initiated. Then, the quenched samples were centrifuged and filtered to separate the solid and liquid. The supernatants were collected and analyzed for the concentration of PhOH using UHPLC (1290 Infinity, Agilent Inc., USA) with a C18 column and an acetonitrile/water (containing 0.1% formic acid) mixture as the mobile phase. For the PhOH measurements, the solvent ratio of the mixture was 20:80 (v:v), and the detection wavelength was 270 nm. For the 2,6-M-PhOH measurements, the ratio was 25:75 and the wavelength was 273 nm.

TOC and COD measurements

In the low-organic-concentration system, 10 mL of suspension/solution was withdrawn from the reaction system at given time intervals and immediately filtered with a 0.22-μm PTFE syringe filter. Then, the filtrate was quenched with sodium sulfite and analyzed for the TOC concentration using a TOC analyzer (Muti N/C 2100, Analytik Jena AG, Germany).

Since mineralization is negligible at low oxidant dosages (a low electron-transfer amount in MnO_X/PhOH systems), the DOTP ratio could be calculated using the following formula: the removal efficiency of aqueous TOC/the removal efficiency of pollutant. Removal efficiency was defined as $(C_0\text{-}C)/C_0$. The DOTP ratio is defined as the proportion of the pollutant reaction accomplished via the DOTP reaction process relative to the total pollutant reaction. In addition, the DOTP ratios in the Mn_3O_4/PhOH, Mn_2O_3/PhOH, and MnO_2/PhOH reaction systems could be verified by calculating the carbon balance using TGA results (i.e., the mass loss on the surface of reacted MnO_X/the mass of the initial PhOH in suspension).

In the high-organic-concentration reaction system, the COD of the solution was measured. Briefly, 3 mL of the suspension was withdrawn at given time intervals and immediately filtered with a 0.22-μm PTFE syringe filter. Then, 2 mL of the filtrate was used as the COD sample and

mixed with COD reagents comprising 1 mL of $K_2Cr_2O_7$ solution (0.16 M, in 10 v/v% H_2SO_4/H_2O), 0.5 mL of $HgSO_4$ solution (100 g/L, in 10 v/v% H_2SO_4/H_2O), and 4 mL of Ag_2SO_4 solution (10 g/L, in concentrated H_2SO_4). Subsequently, the mixtures were heated at 165 °C for 30 min and cooled to ambient temperature. Finally, the obtained clear solutions were analyzed using a UV-visible spectrophotometer (2450, Shimzdzu Co., Japan) at 440 nm.

Structural characterizations

The crystal structure and morphology of the catalysts were characterized using a X-ray powder diffraction spectrometer (XRD, TTR-III diffractometer, Rigaku Co., Japan) with Cu *K*α radiation (λ = 1.5418 Å) and TEM (H7650, Hitachi Co., Japan). The chemical compositions and valence states of constituent elements were analyzed using XPS (ESCALAB 250, Thermo Fisher Inc., USA) with an Al *K*α radiation source, and the binding energy was calibrated with the C-1s peak at 284.8 eV. The mass of the products accumulated on the catalyst was analyzed using TGA spectroscopy (TGA-FTIR, TL-9000, Perkin Elmer Co., USA). The concentrations of the dissolved/leached manganese ions were analyzed using an inductively coupled plasma-mass spectrometer (ICP–MS, PlasmaQuad 3, Thermo Fisher Inc., USA).

LC–MS measurement

The products in the reaction solution or dissolved in ethanol were analyzed using liquid chromatography–mass spectrometry (LC-MS, Orbitrap Exploris 240, Thermo Fisher Inc., USA) equipped with a UHPLC system and a heated electron spray ionization source. For analyses in positive ionization mode, an acidic C18 column (ZORBAX RRHD StableBond C18, 2.1 × 50 mm, 1.8 μm particle size) was used for UHPLC separation. A mixture of acetonitrile and H_2O (acidic, containing 0.1% formic acid) was used as the mobile phase, and the flow rate was set at 0.4 mL min^{-1}. For analyses in negative ionization mode, an alkaline C18 column (ZORBAX RRHD Extend-C18, 2.1 × 50 mm, 1.8 μm particle size) was used for UHPLC separation. A mixture of acetonitrile and H_2O (alkaline, containing 5 mM ammonium hydroxide) was used as the mobile

phase, and the flow rate was set at 0.4 mL min^{-1}. In the separation procedure, a gradient elution method was used, which increased linearly from 10:90 (acetonitrile : H_2O) to 90:10 from 0.5 min to 4.5 min, kept at 90:10 for 1 min, returned to 10:90 in the last 0.5 min and was maintained for 1 min to enable re-equilibration. In addition, an isocratic elution method was also used with a ratio of acetonitrile to water of 50:50 (v:v). The mass calibration range was between 50 and 1200 Da, and the resolution was always kept above 30000.

Gel permeation chromatograph (GPC) measurement

The products dissolved in toluene were dried at 110 °C for 24 h in an oven to remove the solvent, producing a brown soft solid. Part of the soft solid was dissolved in tetrahydrofuran, and the molecular weights (including Mp, Mn, and Mw) and polymer dispersity index were measured using a GPC (150C, Waters Inc., USA) equipped with microstyragel columns and an RI 2414 detector at 35 °C. Tetrahydrofuran with a flow rate of 1.0 mL/min was used as the eluent. The molecular weights were calibrated against monodispersed polystyrene standards.

Matrix-assisted laser desorption/ionization time-of-flight mass spectrometry (MALDI-TOF MS) measurement

The polymeric unit of the soft solid dissolved in tetrahydrofuran was analyzed using a MALDI-TOF MS (Atouflex Speed, Bruker Inc., USA).

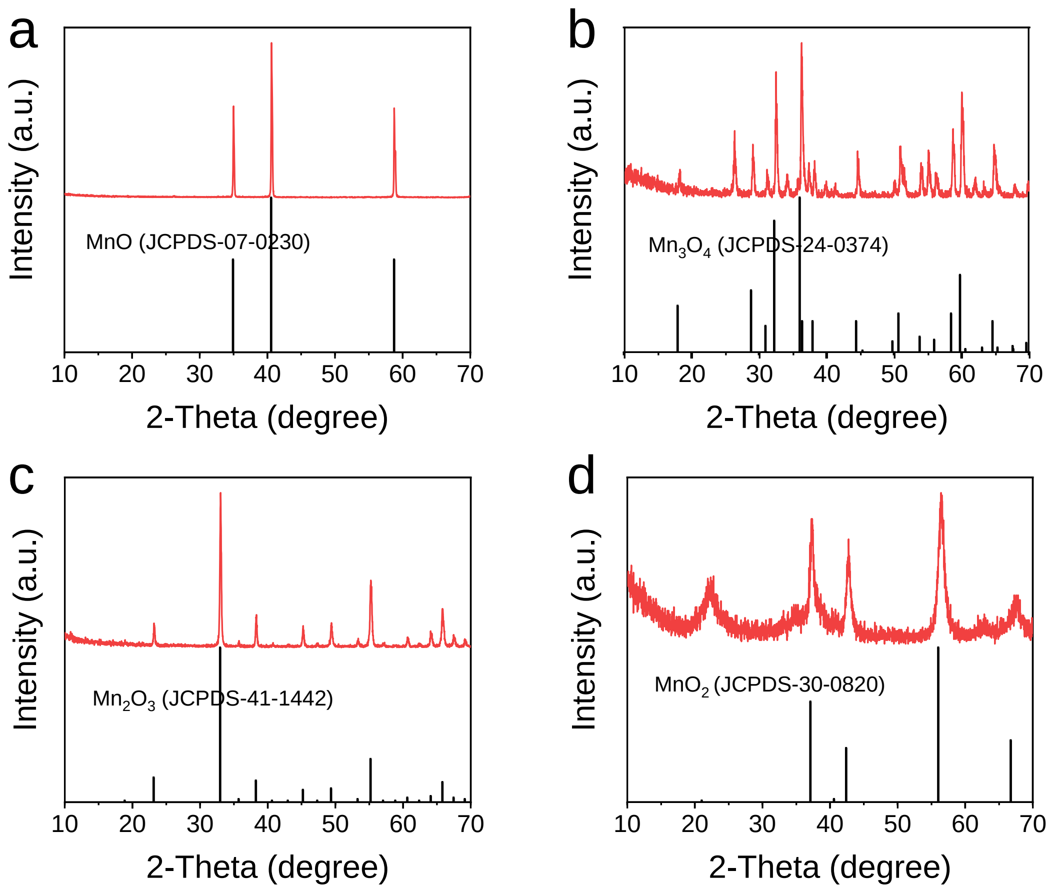

Fig. S1. a, b, c, d, XRD patterns of commercial (**a**) MnO, (**b**) Mn_3O_4, (**c**) Mn_2O_3, and (**d**) MnO_2.

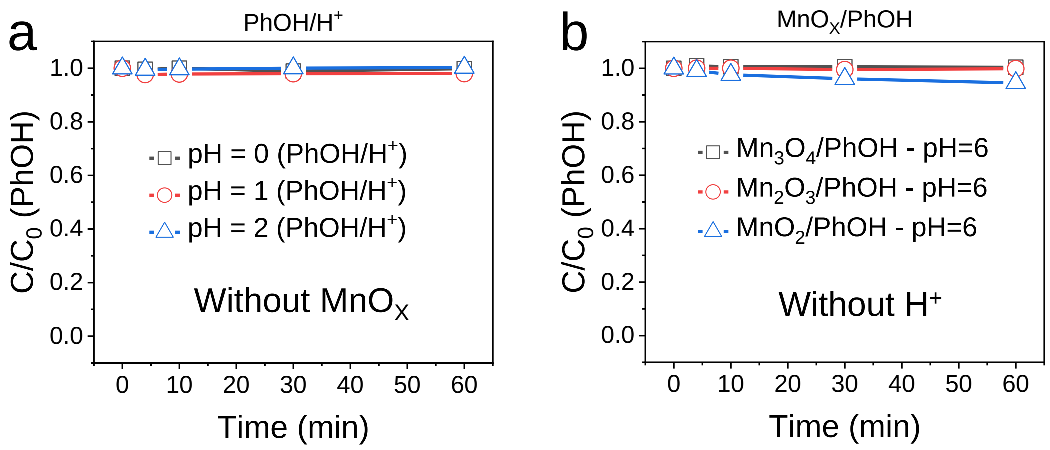

Fig. S2. a, PhOH removal under acidic conditions without MnO_X. **b,** PhOH removal under neutral conditions with MnO_X. ([PhOH] = 12.5 mg L^{-1}, [MnO_X] = 0.2 g L^{-1})

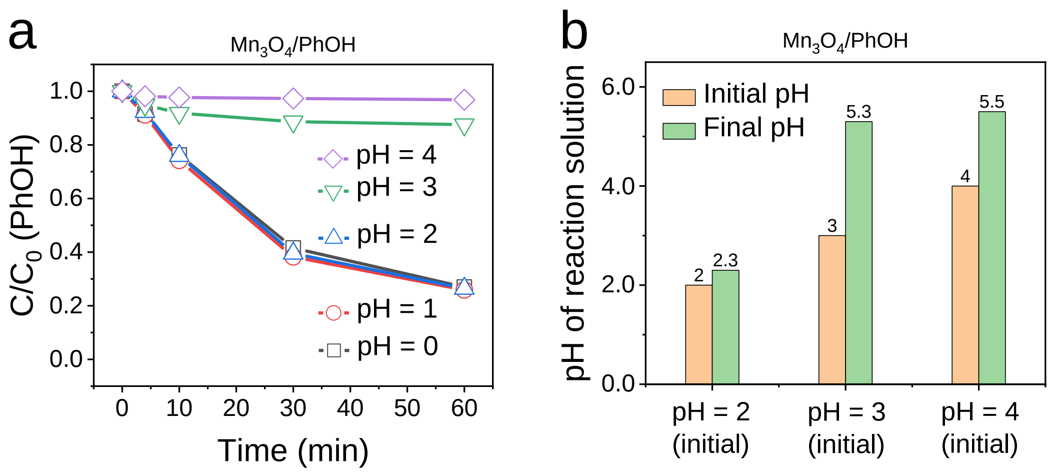

Fig. S3. a, PhOH removal efficiencies in the Mn_3O_4/PhOH system under different pH conditions. **b,** pH changes of the reaction solution in **a**. ([PhOH] = 50 mg L^{-1}, [Mn_3O_4] = 0.2 g L^{-1})

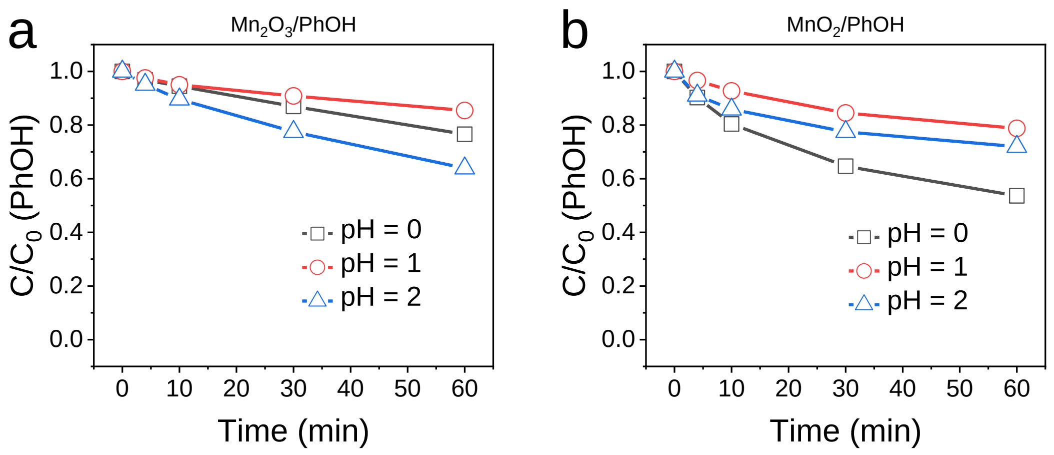

Fig. S4. a, b, PhOH removal efficiencies in the (**a**) Mn_2O_3/PhOH and (**b**) MnO_2/PhOH systems under different pH conditions. ([PhOH] = 50 mg L^{-1}, [Mn_2O_3] = [MnO_2] = 0.2 g L^{-1})

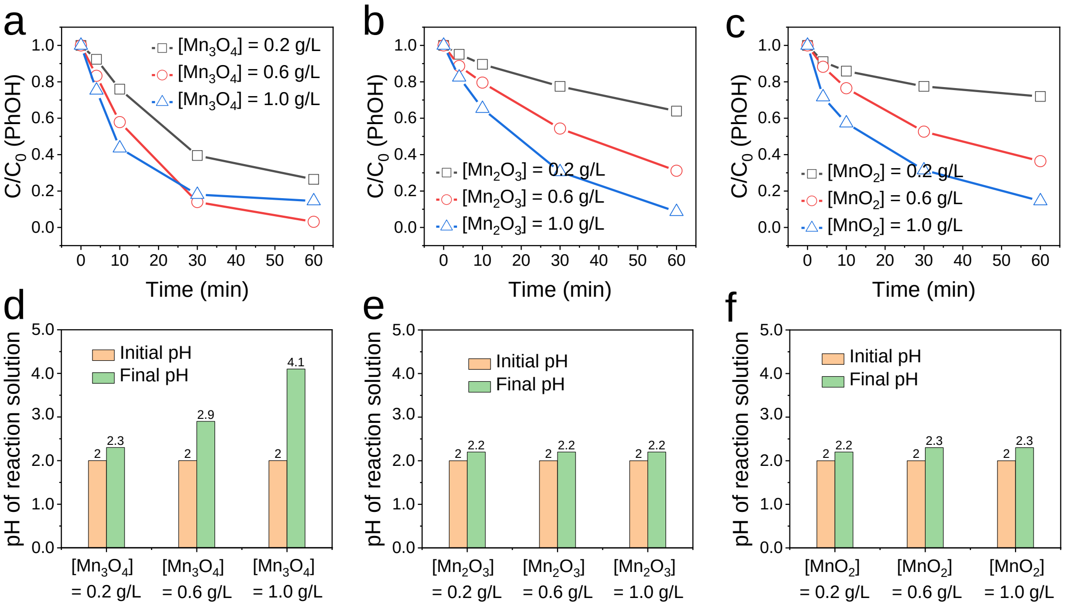

Fig. S5. a,b,c,d,e,f Effect of MnO_X dosages on the PhOH removal efficiencies and pH changes of the reaction solution in the (**a** and **d**) Mn_3O_4/PhOH, (**b** and **e**) Mn_2O_3/PhOH, and (**c** and **f**) MnO_2/PhOH systems. ([PhOH] = 50 mg L^{-1}, pH = 2, [MnO_X] = 0.2 or 0.6 or 1.0 g L^{-1})

In the oxidation reaction, to obtain 2 moles of electrons, Mn_3O_4 will release 3 moles of Mn^{2+} and 4 moles of O, consuming 8 moles of H^+. For Mn_2O_3 and MnO_2, only 2 moles of Mn^{2+}, 3 moles of O and 1 mole of Mn^{2+}, 2 moles of O will be released, consuming 6 and 4 moles of H^+, respectively. Therefore, Mn_3O_4 consumed more H^+ and caused a more pronounced pH change than Mn_2O_3 and MnO_2 under the same conditions.

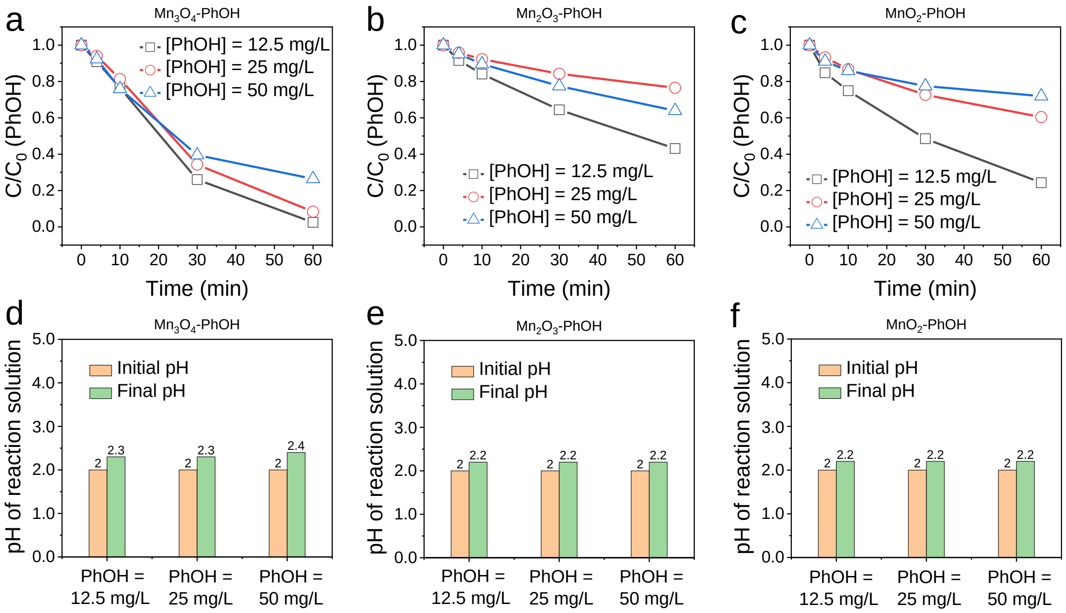

Fig. S6. a,b,c,d,e,f Effect of the initial PhOH concentrations on the PhOH removal efficiencies and pH changes of the reaction solution in the (**a** and **d**) Mn_3O_4/PhOH, (**b** and **e**) Mn_2O_3/PhOH, and (**c** and **f**) MnO_2/PhOH systems. ([PhOH] = 12.5 or 25 or 50 mg L^{-1}, pH = 2, [MnO_X] = 0.2 g L^{-1})

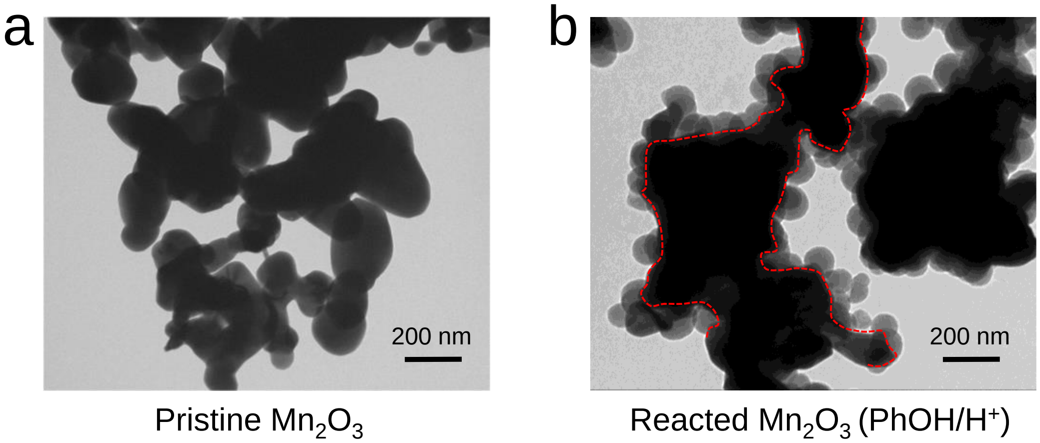

Fig. S7. a,b, TEM images of the (**a**) pristine Mn_2O_3 and (**b**) reacted Mn_2O_3 (Mn_2O_3/PhOH, pH = 2.0).

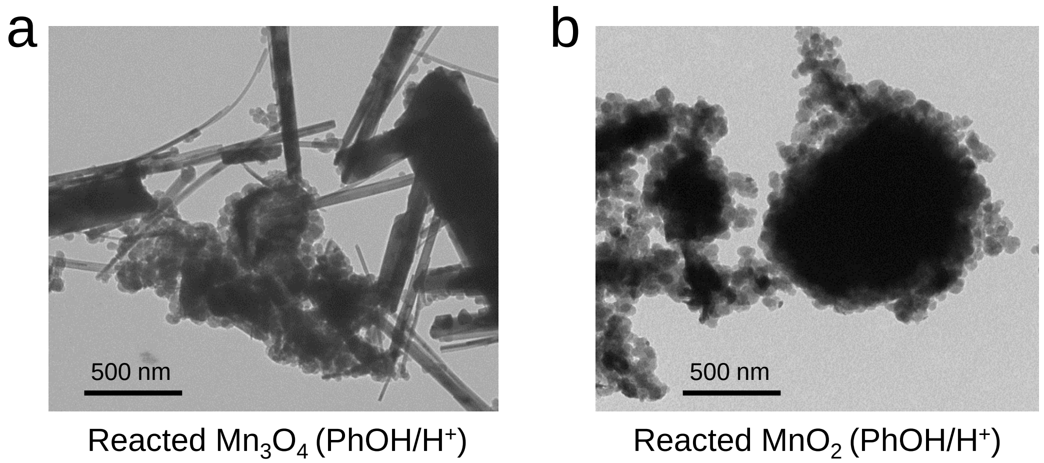

Fig. S8 a,b, TEM images of the reacted (**a**) Mn_3O_4 (Mn_3O_4/PhOH, pH = 2.0) and (**b**) MnO_2 (MnO_2/PhOH, pH = 2.0).

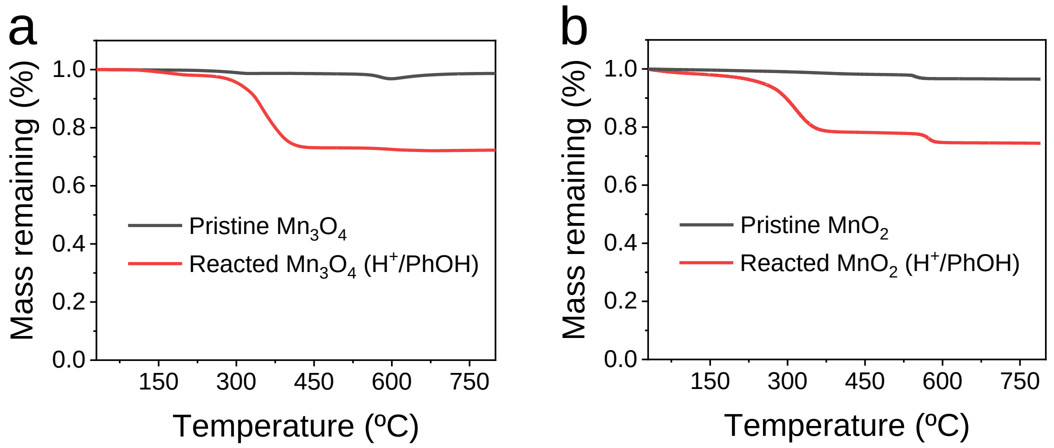

Fig. S9. a, b, TGA curves of the pristine and reacted (**a**) Mn_3O_4 and (**b**) MnO_2 in air (O_2).

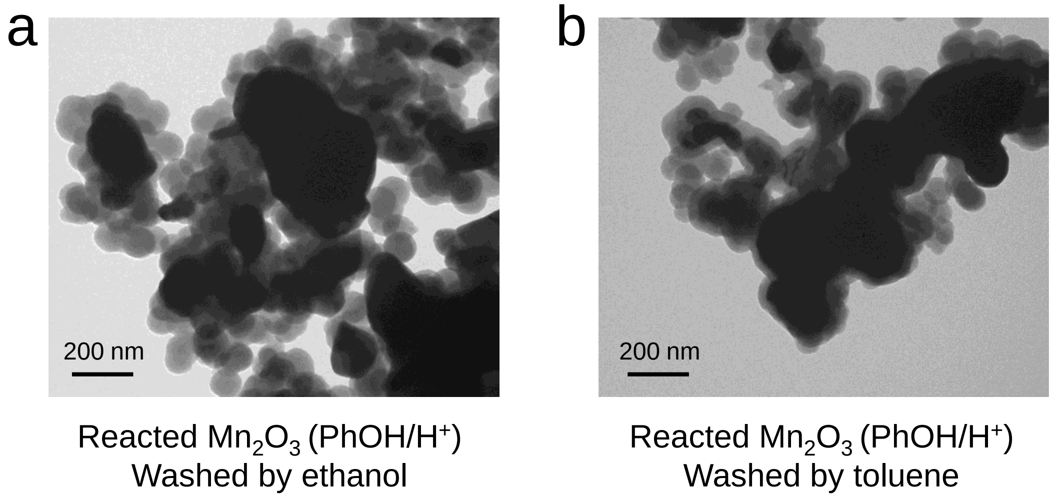

Fig. S10. a, b, TEM images of the reacted Mn_2O_3 (Mn_2O_3/PhOH, pH = 2.0) after elution with (**a**) ethanol and (**b**) toluene.

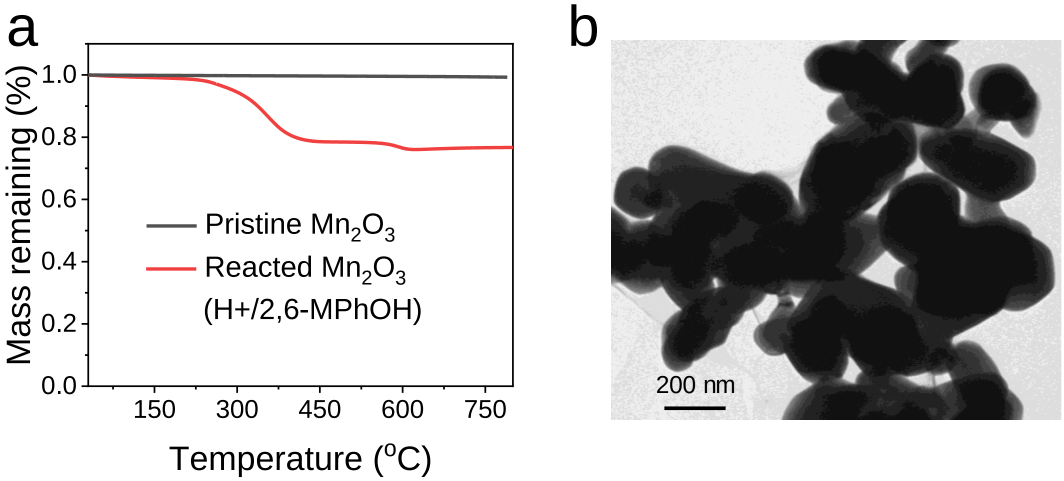

Fig. S11. a, TGA curves of the pristine and reacted Mn_2O_3 (Mn_2O_3/2,6-MPhOH, pH = 2.0) in air (O_2). **b,** TEM image of the reacted Mn_2O_3 (Mn_2O_3/2,6-MPhOH, pH = 2.0) after elution with ethanol and toluene (mixture).

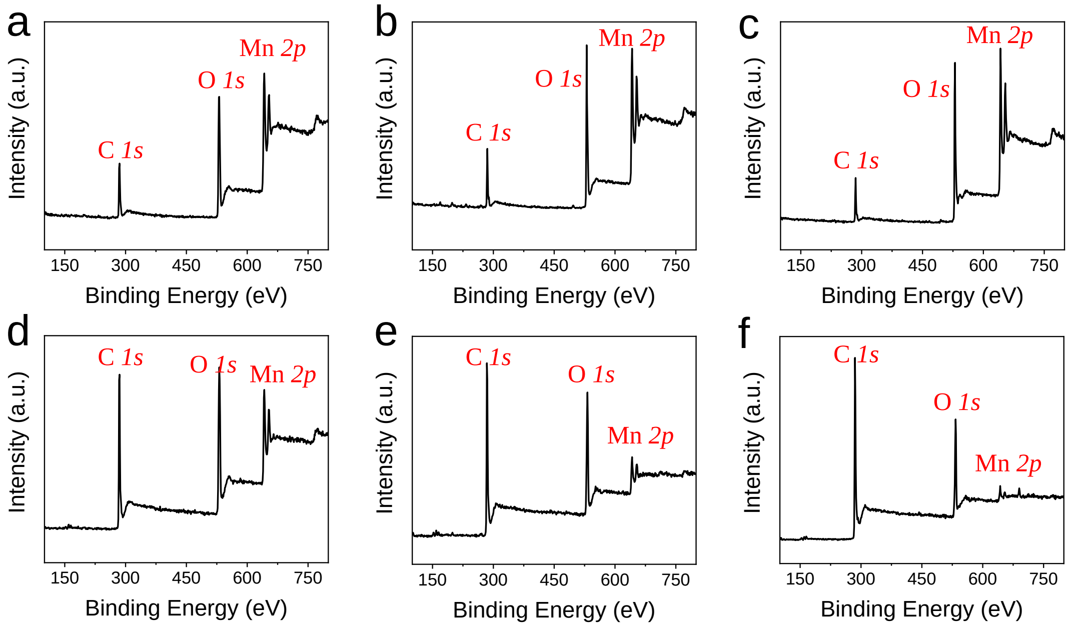

Fig. S12. a, b, c, d, e, f X-ray photoelectron spectroscopy surveys of pristine (**a**) Mn_3O_4, (**b**) Mn_2O_3, and (**c**) MnO_2 and reacted (**d**) Mn_3O_4, (**e**) Mn_2O_3, and (**f**) MnO_2 (MnO_X/PhOH, pH = 2.0)

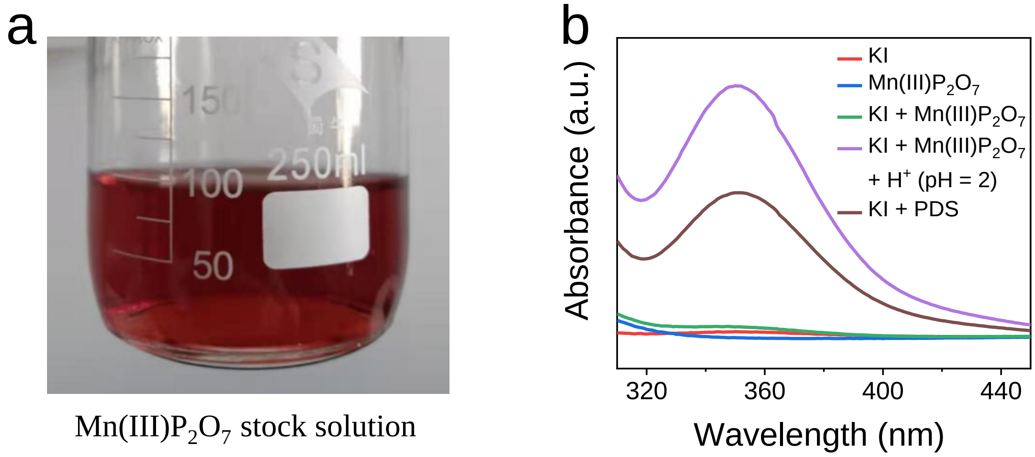

Fig. S13. a, Optical photograph of the Mn(III)P_2O_7 stock solution. **b,** Ultraviolet–visible absorption spectra of the KI oxidation reaction for Mn^{3+} quantitation. PDS, peroxydisulfate.

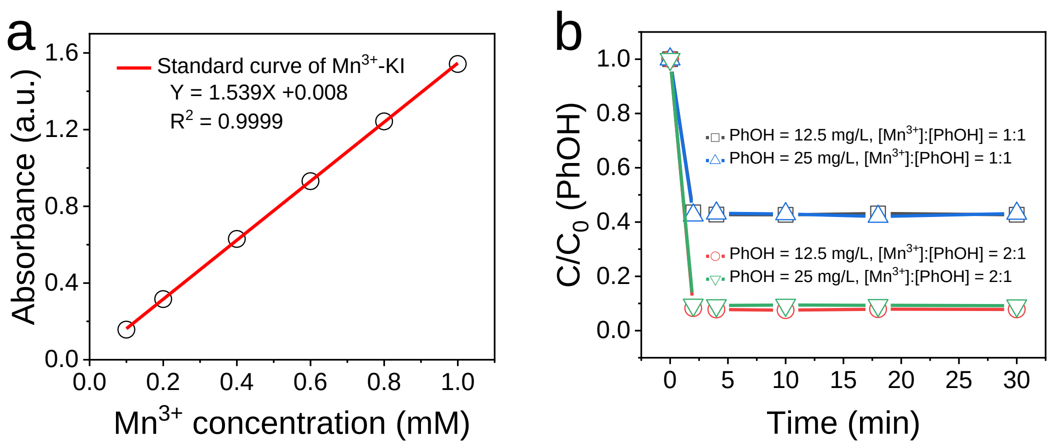

Fig. S14. a, Standard curve for Mn^{3+} quantitation by KI spectrophotometry (recording wavelength was set to 396 nm). **b,** PhOH removal efficiency at different dosages of PhOH and Mn^{3+}.

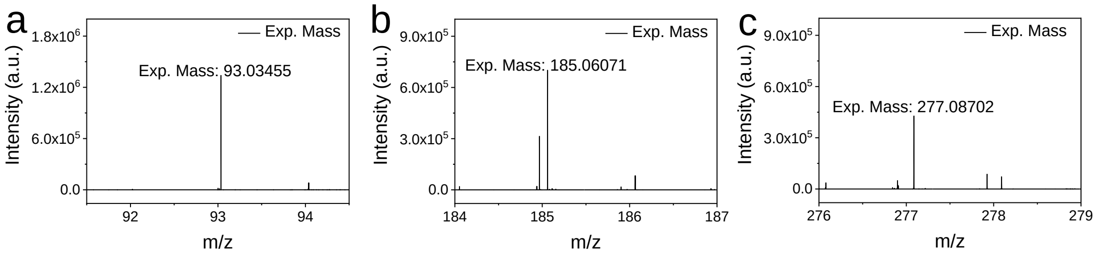

Fig. S15. a, b, c, MS spectra of (**a**) PhOH, (**b**) dimers of PhOH, and (**c**) trimers of PhOH in the Mn^{3+}-dominated PhOH oxidative removal system. Exp., experimental.

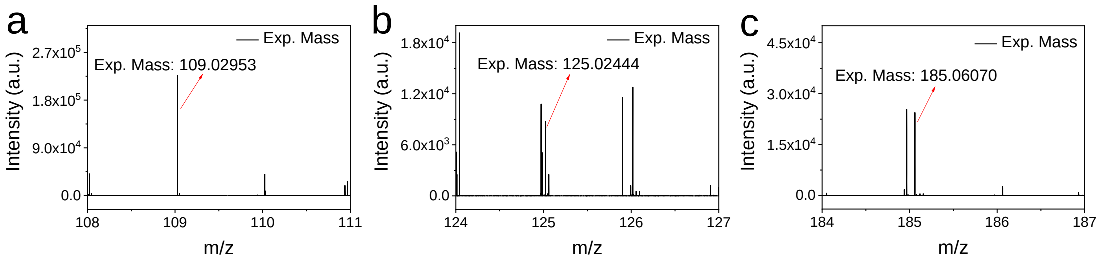

Fig. S16. a,b,c MS spectra of (**a**) benzenediols, (**b**) benzenetriols, and (**c**) dimers of PhOH in the •OH-dominated PhOH oxidative removal system. Exp., experimental.

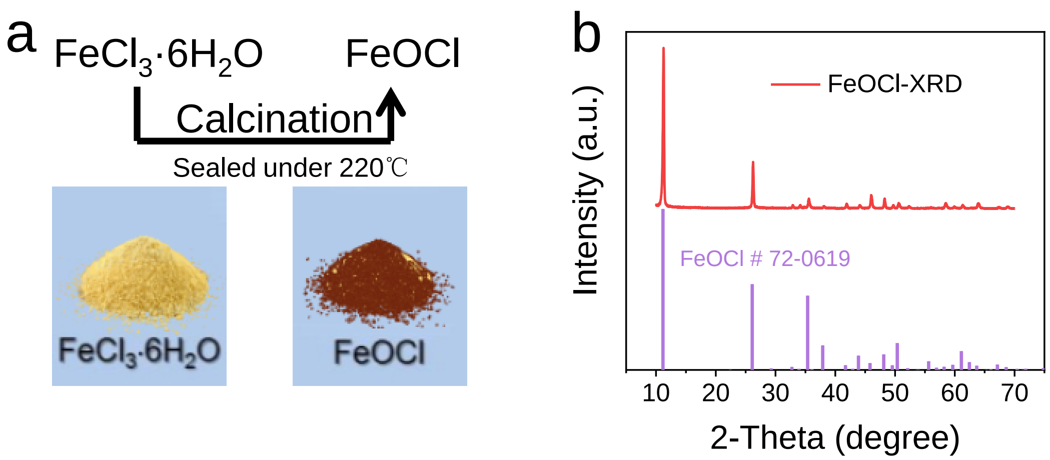

Fig. S17. a, Process of FeOCl preparation. **b,** XRD pattern of the prepared FeOCl.

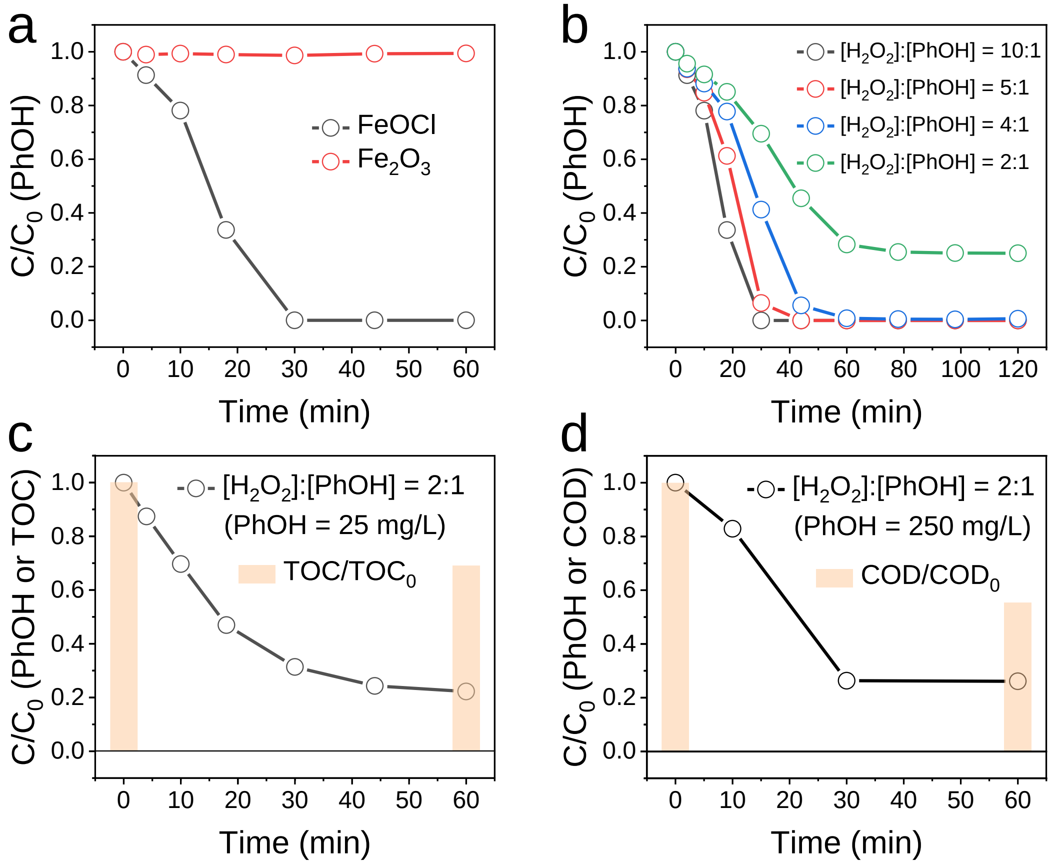

Fig. S18. a, PhOH removal in the H_2O_2 oxidation system with FeOCl and Fe_2O_3 catalysts under the same reaction conditions ([PhOH] = 12.5 mg L^{-1}, [FeOCl] = [Fe_2O_3] = 0.2 g L^{-1}, [H_2O_2]:[PhOH] = 10:1). **b,** PhOH removal efficiencies in the FeOCl/H_2O_2/PhOH system under different dosage ratios of H_2O_2 to PhOH. **c,** Removal efficiencies of PhOH and TOC at a 2:1 dosage ratio of H_2O_2 to PhOH (low concentration system, [PhOH] = 25 mg L^{-1}, [FeOCl] = 0.2 g L^{-1}). **d,** Removal efficiencies of PhOH and COD at a 2:1 dosage ratio of H_2O_2 to PhOH (high concentration system, [PhOH] = 250 mg L^{-1}, [FeOCl] = 1.0 g L^{-1})

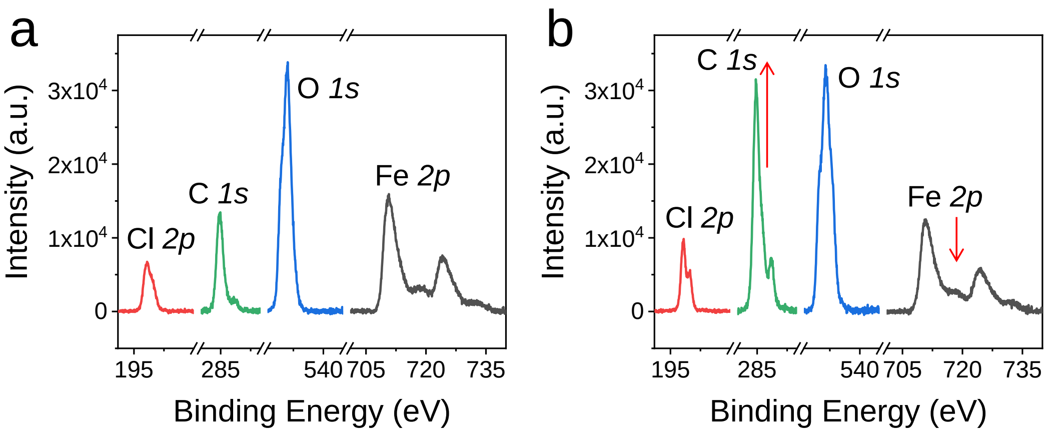

Fig. S19. a, b, XPS spectra of (**a**) pristine FeOCl and (**b**) reacted FeOCl (FeOCl/H_2O_2/PhOH).

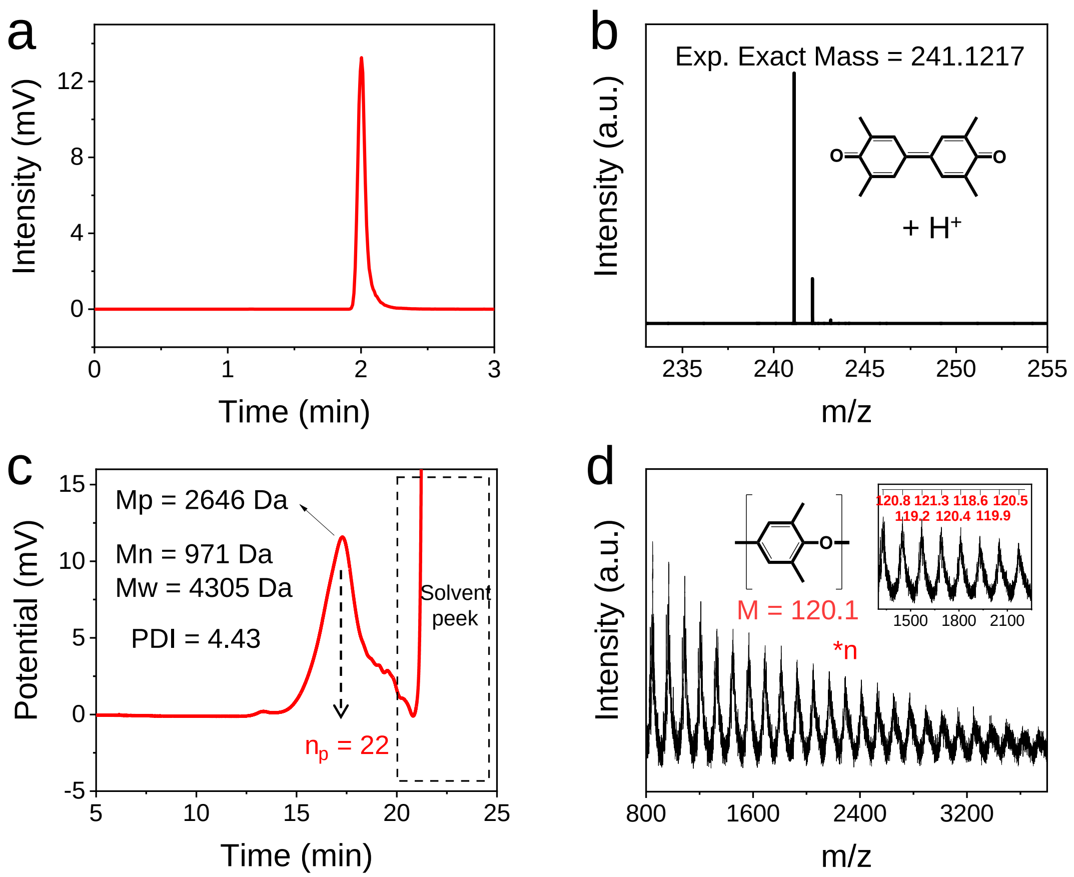

Fig. S20. a,b, High-performance liquid chromatogram (**a**) and high-resolution mass spectrum (**b**) of the products on the reacted FeOCl (FeOCl/H_2O_2/2,6-PhOH) washed off by ethanol. **c, d,** Gel permeation chromatogram (**c**) and MALDI-TOF-MS spectrum (**d**) of the products on the reacted FeOCl (FeOCl/H_2O_2/2,6-PhOH) washed off by toluene. Exp., experimental; Mp, peak molecular weight; Mn, number average molecular weight; Mw, weight average molecular weight; N, degree of polymerization.

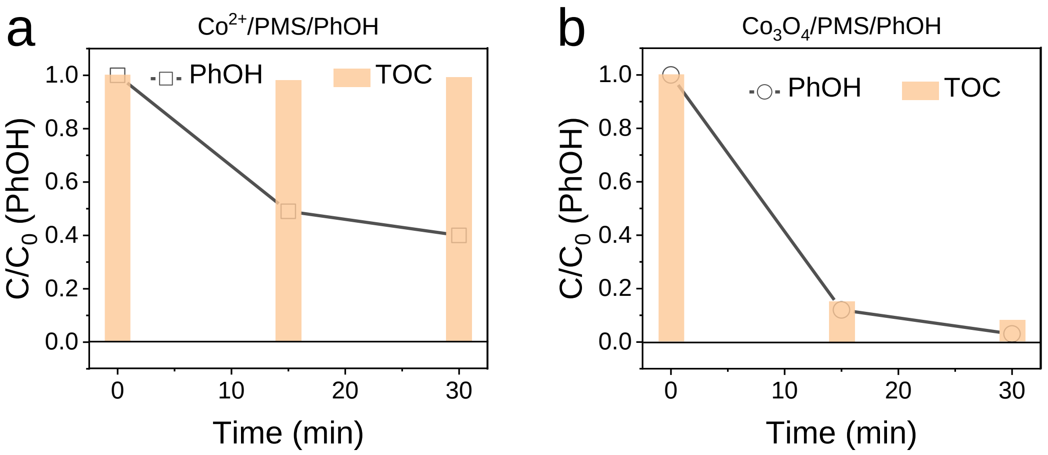

Fig. S21. a, b, Removal behaviors of aqueous PhOH and TOC in the (**a**) Co^{2+}/PMS/PhOH homogeneous catalytic oxidation system and (**b**) Co_3O_4/PMS/PhOH heterogeneous catalytic oxidation system. ([PhOH] = 25 mg L^{-1}, [$CoSO_4$] = 0.1 mM, [Co_3O_4] = 0.2 g L^{-1}, [PMS]:[PhOH] = 2:1)

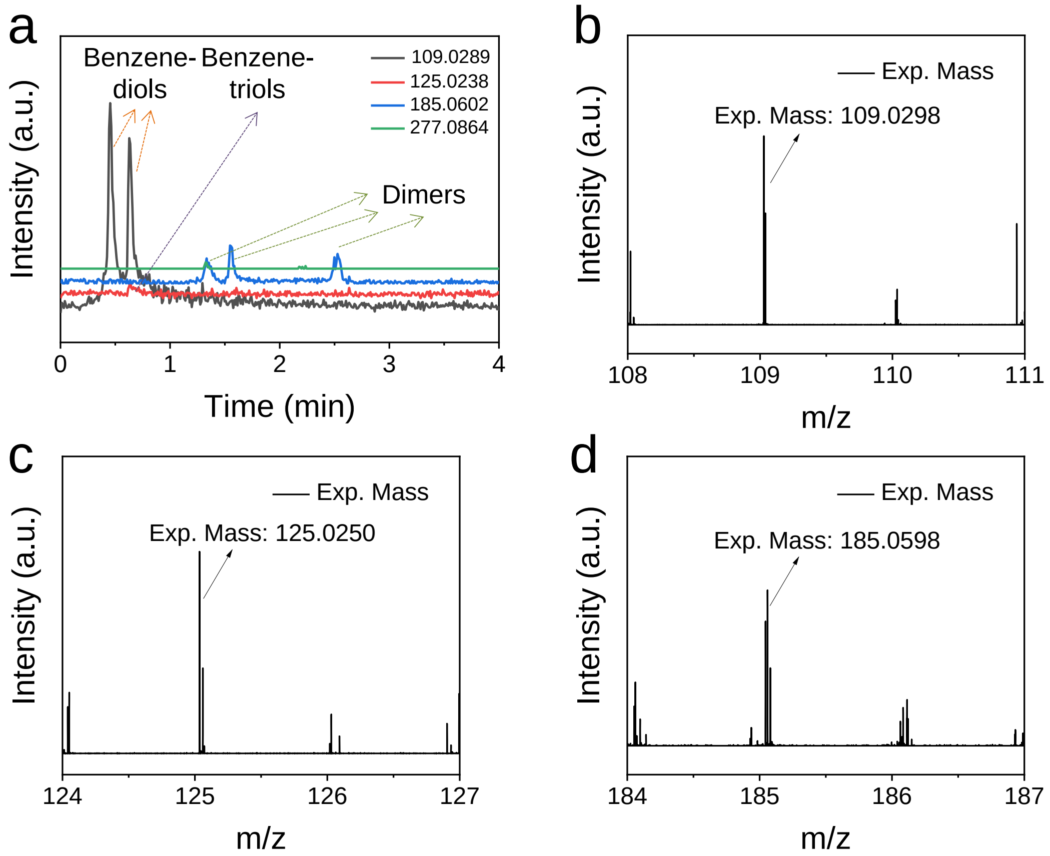

Fig. S22. a, Products separation and identification in the sulfate-radical dominated Co^{2+}/PMS/PhOH homogeneous oxidation system by LC-MS. **b,c,d,** MS spectra of (**b**) benzenediols, (**c**) benzenetriols, and (**d**) dimers of PhOH in **a**.

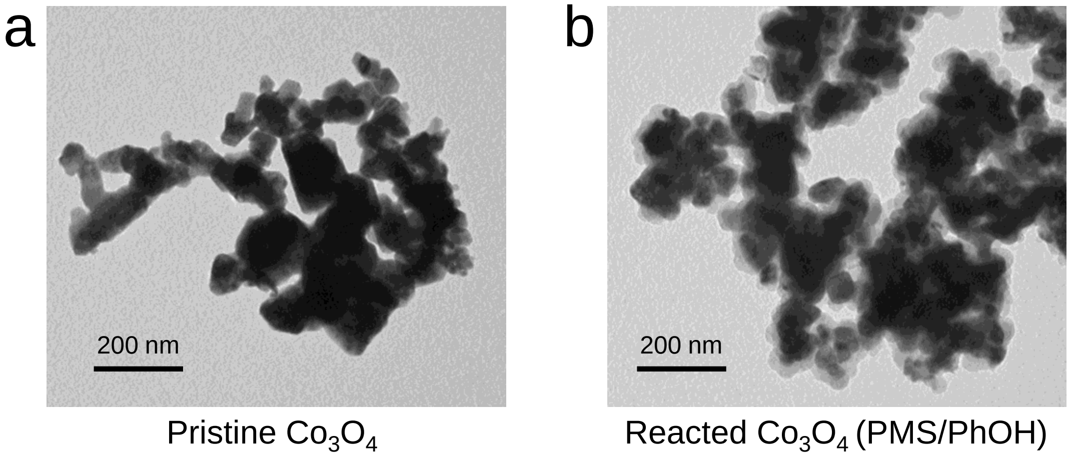

Fig. S23. a,b, TEM images of the (**a**) pristine Co_3O_4 and (**b**) reacted Co_3O_4 (Co_3O_4/PMS/PhOH).

Supplementary Table 1. Leached Mn ions at pH 0.7 in the MnO_X/H_2SO_4 systems after 1-h mixing of MnO_X and H_2SO_4 measured by ICP–MS.

MnO_X: 0.2 g L^{-1} H_2SO_4: 0.1 M	MnO + H_2SO_4	Mn_3O_4 + H_2SO_4	Mn_2O_3 + H_2SO_4	MnO_2 + H_2SO_4
Mn leaching amount (mg L^{-1})	197.91	0.13	0.08	0.19

Supplementary Table 2. Leached Mn ions at pH 2.0 after 1-h mixing of MnO_X and H_2SO_4 measured by ICP–MS.

MnO_X: 0.2 g L^{-1}<br>H_2SO_4: 0.005 M	Mn_3O_4<br>+ H_2SO_4	Mn_2O_3<br>+ H_2SO_4	MnO_2<br>+ H_2SO_4
Mn leaching amount (mg L^{-1})	0.11	0.09	0.28
